# Molecular Drivers of Aging in Biomolecular Condensates: Desolvation, Rigidification, and Sticker Lifetimes

**DOI:** 10.1103/prxlife.2.023011

**Published:** 2024-06-06

**Authors:** Subhadip Biswas, Davit A. Potoyan

**Affiliations:** 1 Department of Chemistry, Iowa State University, Ames, Iowa 50011, USA; 2 Department of Biochemistry, Biophysics and Molecular Biology, Iowa State University, Ames, Iowa 50011, USA

## Abstract

Biomolecular condensates are dynamic intracellular entities defined by their sequence- and composition-encoded material properties. During aging, these properties can change dramatically, potentially leading to pathological solidlike states, the mechanisms of which remain poorly understood. Recent experiments reveal that the aging of condensates involves a complex interplay of solvent depletion, strengthening of sticker links, and the formation of rigid structural segments such as beta fibrils. In this study, we use various coarse-grained models to investigate how solvent expulsion, biopolymer chain rigidity, and the lifetimes of sticker contacts influence the viscoelastic properties and aging dynamics of condensates. We find that the rigidity of the biopolymer backbone is essential for replicating the predominant elastic behavior observed in experiments. In contrast, models using fully flexible chains—an assumption common in simulations of intrinsically disordered proteins—fail to exhibit a dominant elastic regime. We also demonstrate that altering the solvent content within condensates affects the crossover between storage and loss moduli. This suggests that desolvation plays a significant role in condensate aging by promoting the transition from a viscous to an elastic state. Furthermore, the lifetime of sticker pairs profoundly influences the mature state of the condensates; short-lived stickers lead to a Maxwell fluid behavior, while longer-lived, irreversibly cross-linked stickers result in solidlike properties, consistent with the Kelvin-Voigt model. Finally, by incorporating the chain rigidification, desolvation, and sticker pair formation into a nonequilibrium dynamic aging simulation, we show the molecular mechanism of forming solid shells around the condensate surfaces observed in a recent experimental report.

## I. INTRODUCTION

**FIG. 1.**
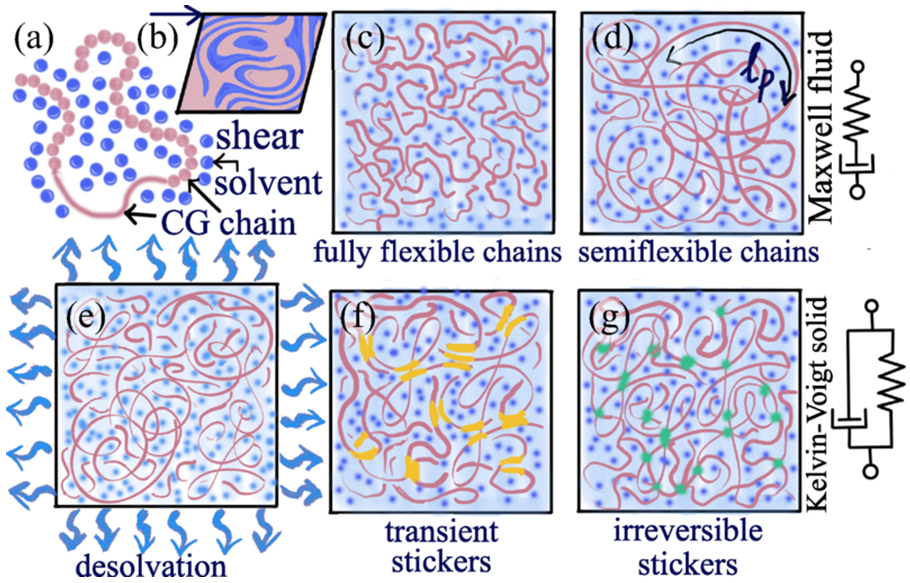
The schematic of models used to dissect the molecular driving forces of aging in biomolecular condensates. (a) A coarse-grained protein model with an explicit solvent is used to determine the rheological properties of condensates using (b) oscillatory shearing of the simulation box. (c) Fully flexible and (d) semiflexible chain models are used to analyze the role of chain rigidity on viscoelastic properties. (e) Solvent expulsion-driven shrinking is modeled in nonequilibrium simulations with (f) short-lived thermoreversible sticker pairs and (g) long-lived sticker pairs forming gel-like condensates.

Biomolecular condensates, formed through the phase separation of proteins and nucleic acids, have emerged as a paradigm for understanding numerous cellular functions [1-7]. A key physical characteristic underlying the proposed functions of biomolecular condensates is their liquidlike behavior, which can be readily tuned by environmental and biochemical perturbations [8-11]. Recent microrheology measurements, however, have shown that condensates are better described as complex viscoelastic fluids that can behave from highly viscous to highly elastic entities depending on sequence [12,13]. Over time, material condensate can age with material properties evolving from liquidlike to solidlike, a phenomenon which has been linked with the onset of neurodegenerative diseases [14-23]. Condensate aging has many parallels to aging in soft glasses, characterized by a relaxation time that increases with time [15,24-29]. In particular, the mean-field and soft glassy rheology models explain the rheological features of aging where the time-dependent changes in their material properties by introducing interaction well between sticker molecules [7,30,31]. Previous computer simulations of nonequilibrium condensate aging [32,33] have demonstrated that changing the rate of protein-protein interaction strength has a significant impact on maturation dynamics and can induce the formation of multiphase architectures from single-component condensate phases. Recent experiments, however, have shown that the aging of condensates involves a complex interplay of solvent depletion, strengthening of sticker links, and the formation of rigid structural segments such as beta fibrils [15-17,34,35]. In this study, we have employed various coarse-grained models to systematically dissect how solvent expulsion, biopolymer chain rigidity, length, and the lifetimes of sticker contacts influence the viscoelastic properties and aging dynamics of condensates (Fig. 1). We report that the condensates’ elastic response domination arises solely from the rigidity of the chains. Meanwhile, fully flexible chains exhibit viscous dominance throughout the frequency range. The Maxwell fluidic nature of the biomolecular condensates, absent in the previous simulations studies, emerges due to the introduction of angular potentials in the chains. Similar rheological phenomena are also observed using a sequence-specific FUS and PGL-3 proteins model. We show solvent expulsion plays a crucial role in aging by bringing sticky regions closer, shifting from a fluidic to elastic nature in the intermediate deformation frequencies.

Lastly, we have incorporated the key molecular processes associated with aging—chain rigidity, desolvation, and sticker pair formation—into a nonequilibrium dynamic simulation to model the aging process explicitly. An intricate interplay among these molecular events collectively contributes to the formation of a solidlike shell around the condensate surface. This shell formation and solvent entrapment within aged condensates align well with recent experimental reports [34]. Our simulations also demonstrate that due to the formation of the rigid shell at the corona region, a significant solvent fraction remains trapped within the condensate, consistent with findings from recent experiments [15-17,34,35]. We believe that through this nonequilibrium mechanistic exploration of aging, we offer a comprehensive mechanistic insight into the molecular underpinnings of aging in condensates, paving the way for further exploration in this field.

## II. MODELS AND METHODS

### A. Sticker-spacer models with explicit solvent

**FIG. 2.**
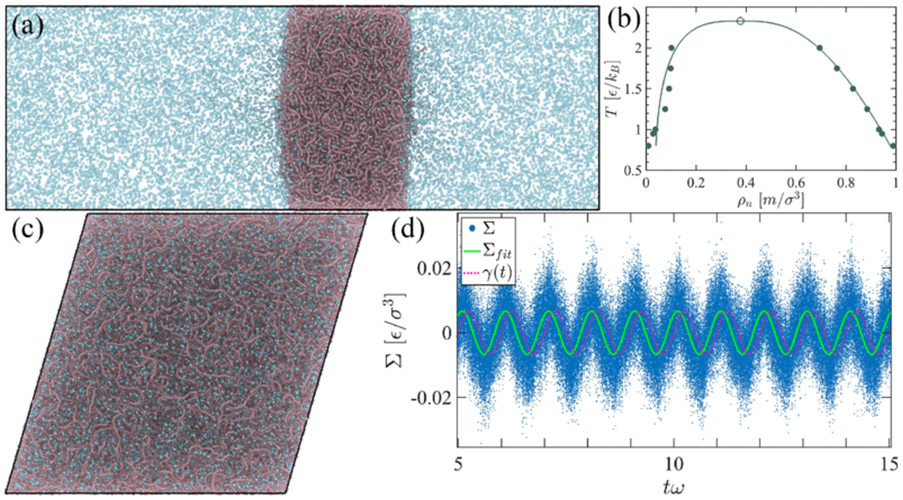
(a) Simulation snapshot of the condensed slab of the coexisting polymer-solvent model where polymer beads are depicted in pale chestnut color and solvent beads are shown in turquoise. (b) Phase diagram of the solvent-polymer mixture as a function of temperature. The critical values are calculated by extrapolating data fitting, giving $T_{c}=2.4\epsilon ∕kT$ and $\rho_{c}=0.38$. (c) A simulation snapshot of the condensed polymer bulk phase in a cubic box shared along the xy direction. (d) An oscillatory shear strain (dotted line) is applied to the simulation box to deform it. The resulting stress data has been generated due to the applied strain, $\Sigma_{xy}$, shown by blue dots. Fitting the stress data is represented by the solid green line.

We have employed generic sticker-spacer models with an explicit solvent to dissect the contributions of chain rigidification (mimicking beta-strand formation) and sticker-pair formation and desolvation (mimicking solvent flow driven by sticker pair formation). In sticker-spacer models, protein chains are connected by anharmonic FENE springs described by the potential $U_{\text{FENE}}(r)=-\frac{1}{2}kR_{0}^{2}\text{ln}(1-r^{2}∕R_{0}^{2})$, where r represents the distance between adjacent beads, a spring coefficient of $k=30\varepsilon ∕\sigma^{2}$, a maximum spring extensibility of $R_{0}=1.5\sigma$ is used and σ is the bead diameter sets the length unit. Bond angles between three consecutive beads are fixed by the angular harmonic potential $U_{\theta }=K_{\theta }{\left(\theta -\theta_{0}\right)}^{2}$, where $K_{\theta }$ is the potential energy and equilibrium angle is $\theta_{0}$. Non-bonded interactions are introduced by Lennard-Jones (LJ) potential (repulsive and attractive), $U_{\text{LJ}}(r)=4\varepsilon [{\left(\sigma ∕r\right)}^{12}-{\left(\sigma ∕r\right)}^{6}]$, which applies when considering purely repulsive interaction $r\leq r_{c}$, and $U_{\text{LJ}}(r)=0$ otherwise. Solvent particles are introduced by adding such nonbonded repulsive monomers in the system. On the other hand, an attractive part can also be implemented by making the cutoff distance $r_{c}$ larger than bead diameter σ, which typically we set $r_{c}=4\sigma$ where potential goes to zero. To avoid the sudden jump in the interaction component of the Lennard-Jones (LJ) potential at the cutoff distance, a linear term is commonly employed to ensure that both the potential energy and the force gradually approach zero [36,37]. Here, ε represents the maximum depth of the interaction energy, and $r_{c}=2^{1∕6}\sigma$ serves as the repulsive cutoff radius. In the simulation of the sticker-spacer models, all quantities are expressed in LJ units, encompassing energy and length parameters (ε and σ, respectively) of the LJ potential, as well as particle mass (m) considered as unity. Thus, the reference units for time, density, temperature, and pressure are given by $\tau^{*}=\sqrt{m\sigma^{2}∕\varepsilon }$, $\rho^{*}=\sigma^{-3}$, $T^{*}=\varepsilon ∕k_{B}$, and $p^{*}=\varepsilon \sigma^{-3}$, respectively. A typical phase separation and solvent-polymer coexistence systems snapshot is shown in Fig. 2(a). The critical point is estimated through extrapolation using rectilinear diameters and the universal scaling of coexistence densities asymptotically close to the critical point, $\frac{\rho_{l}+\rho_{v}}{2}=\rho_{c}+A(T_{c}-T)$ and $\Delta \rho =\Delta \rho_{0}+{\left(1-T∕T_{c}\right)}^{\beta }$ where the exponent is considered as β=0.325 shown in Fig. 2(b).

In the minimal LJ model framework, along with considering the desolvation effect on homopolymer chains, we have also introduced sticker beads to capture other effects, e.g., β-sheet formation that significantly contributes to the aging of these condensates. In order to introduce sticker lifetimes, we use two different approaches: (i) irreversible cross-link of the sticker-sticker beads, and (ii) transient stiff stickers strengthening of interactions between proteins via attractive LJ interaction with ε, which can bind and unbind with the temperature and desolvation. Sticker beads have been introduced at intervals of ten spacer beads. The angle between three consecutive sticker beads is set to $\theta \approx 180^{{}^{\circ}}$, resulting in stiff stickers.

### B. Residue-resolution model with explicit solvent

We utilize the recently reparametrized [38] HPS (hydropathy scale) coarse-grained model [39,40], which is widely used for describing the phase behavior of intrinsically disordered proteins. This coarse-grained model employs one bead per amino acid or nucleotide. The simulations are conducted under NVT ensemble conditions using a Langevin thermostat with a time step of Δt=10fs. We mimic the early condensed phase by adding solvent particles inside the phase-separated FUS protein to establish weak and promiscuous protein-protein interactions at short molecular distances using a short-ranged attractive Lennard-Jones (LJ) potential. Typically, solvent and proteins are located inside the condensate in a colocalized state. However, implementing only the repulsive interaction of the solvent with the protein residue leads to phase separation from the protein. Therefore, a minimal attractive interaction is necessary to ensure homogeneity in the protein and solvent condensate. It is important to note that desolvation does not result from the phase separation between the protein and solvent components, rather, the diffusion process drives it. Thus, desolvation is a slow process.

### C. Rheology of explicitly solvated biomolecular condensates

To study bulk rheological properties, periodic boundary conditions have been employed, NVT ensemble coupled with stochasticlike linear orderly dissipative (SLLOD) algorithm [41] and Langevin thermostat. The SLLOD equations of motion yield the desired velocity gradient and accurately account for work production due to stresses in a wide range of homogeneous flow scenarios. To perform nonequilibrium MD (NEMD) simulations of a continuously strained system, we deform the box along xy direction with sinusoidal strain $\gamma_{xy}=\gamma_{0}$ sin ωt with amplitude $\gamma_{0}$ falls in the linear response regime and oscillation frequency ω [36] [see Fig. 2(c)]. Due to the strain, the stress generated by each system particle $\Sigma_{xy}$ also shows oscillatory behavior with a phase lag of the applied strain, shown in Fig. 2(d). We choose the integration time step of typically Δt=0.01−0.005τ (LJ units) while integrating Newton’s equations of motion. We have also implemented equilibrium molecular dynamics with the Green-Kubo (GK) approach. The multi-τ correlator method effectively reduced noise while preserving accurate relaxation dynamics. From the equilibrium stress autocorrelation function of an isotropic system one can approximate complex modulus as:

(1) G∗(t)=V5kBT[〈Σxy(0)Σxy(t)〉+〈Σyz(0)Σyz(t)〉]+〈Σxy(0)Σxz(t)〉+16(〈Nxy(0)Nxy(t)〉+[〈Nyz(0)Nyz(t)〉+〈Nxz(0)Nxz(t)〉)],

where, $\Sigma_{ij}$ are the off-diagonal component of stress tensor and $N_{ij}=\Sigma_{ii}-\Sigma_{jj}$ are the normal stress tensor difference.

When performing oscillatory shear (OS) simulations, it is crucial to ensure that this strain magnitude remains within the linear viscoelastic regime, where the dynamic moduli do not change significantly with variations in $\gamma_{0}$ (see Supplemental Material, Fig. S1 [42]). In this method, a sinusoidal strain is applied as $\gamma =\gamma_{0}\;\sin(\omega t)$. The resulting stress generated due to this applied strain response, denoted as $\Sigma_{xy}=\Sigma_{0}\;\sin(\omega t+\delta )$, is observed. Here, ω signifies the oscillation frequency, and $\Sigma_{0}$ represents the stress amplitude.

By varying solvent concentrations in bulk condensed systems, we measure the frequency-dependent complex shear modulus $G^{*}=G^{\prime }+iG^{\prime \prime }$ of the bulk system at different solvent fractions. The storage ($G^{\prime }$) modulus represents the elastic response of the condensate, indicating its ability to store energy, while the loss ($G^{\prime \prime }$) modulus signifies the viscous response, exhibiting the dissipation of energy, under deformation. We unveil distinct frequency-dependent regimes in which $G^{\prime }$ and $G^{\prime \prime }$ intersect, and their crossover regions either exhibit liquidlike or solidlike responses, characterizing the condensates.

The complex or dynamic viscosity, denoted as $\eta^{*}$, can be calculated using $\eta^{*}=\int_{0}^{\infty }G^{*}(t^{\prime })dt^{\prime }$. We fit the data with the generalized Maxwell model $G^{\prime }=\sum_{i}G_{i}^{\prime }\frac{\omega_{i}^{2}\tau_{i}^{2}}{1+\omega_{i}^{2}\tau_{i}^{2}}$, $G^{\prime \prime }=\sum_{i}G_{i}^{\prime \prime }\frac{\omega_{i}\tau_{i}}{1+\omega_{i}^{2}\tau_{i}^{2}}$ to infer the ω→0 limit response.

Different simulation techniques (e.g., GK, OS, bead tracking) have been introduced to characterize viscoelastic properties using MD simulations of the HPS protein CG residue model [43]. However, none can capture the different $G^{\prime }$, $G^{\prime \prime }$ (one over other) dominated regions across the frequency window scale.

## III. RESULTS

### A. Role of solvent expulsion

**FIG. 3.**
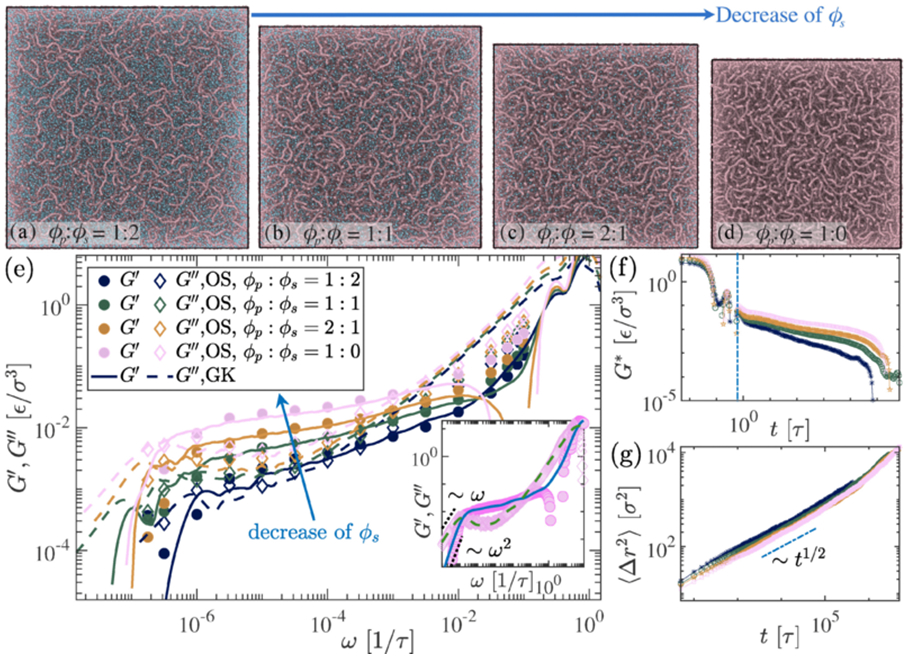
(a)–(d) Simulation snapshots depict polymer fraction $\phi_{p}$ (with fixed $\ell_{p}\approx 5\sigma$, pale chestnut color) and solvent (turquoise beads) fractions $\phi_{s}$ at various concentrations. (e) Viscoelastic properties, quantified by storage ($G^{\prime }$) and loss ($G^{\prime \prime }$), shown for different solvent fractions obtained using Green-Kubo (GK) and oscillatory shear (OS) methods. The inset of (e) displays the fitting of the generalized Maxwell model to $G^{\prime }$ and $G^{\prime \prime }$ from GK data for the scenario where $\phi_{s}=0$. (f) The complex modulus ($G^{*}$) for four different concentrations was obtained using the GK method. The vertical dashed line separates the short and long time ranges, which are used to evaluate the Maxwell modes for longer timescales. (g) MSD of the chains behave subdiffusively as $\langle \Delta r^{2}\rangle ∼t^{1∕2}$.

Viscoelastic responses of the biopolymer solutions are sensitive to the proportion of polymer and solvent fractions. To model the aging dynamics of condensates, which can span time scales of several hours, we instead vary the solvent fraction while keeping the polymer concentration and rigidity ($\ell_{p}\approx 5\sigma$) fixed. We take four different polymer and solvent fractions ($\phi_{p}\;:\phi_{s}$) as 1:2, 1:1, 2:1, and 1:0 [Figs. 3(a)-3(d)], which represent different stages of aging where one sees evolution towards denser condensates. Initially, the condensed phase has a significant presence of solvent molecules; however, over time, the solvent concentration decays due to various processes inside the droplet. The material properties are influenced by the behavior of their constituent molecule properties such as length (N), flexibility ($\ell_{p}$), interaction strengths ($\varepsilon_{ij}$) etc., which we vary to show the distinct rheological properties.

In early times, many biomolecular condensates exhibit liquidlike behavior, indicating solvent-rich conditions. However, as the solvent molecules evaporate with time, the biomolecules within the condensed phase tightly compact due to strong contacts, leading to a transition towards a more solidlike state. We also compare polymeric systems (Maxwell fluid behavior) with the cross-linked polymer gel, giving distinct Kelvin-Voigt solid features in the viscoelastic response. We show the storage and loss modulus of the long chains in different solvent concentrations [Fig. 3(e)]. Both match closely over the wide frequency range (ω) values in the low shear amplitude. It is noted that, in the case of the oscillatory shear (OS) method, only small deformations γ∼0.1, i.e., in the linear viscoelastic regime, $G^{\prime }$ and $G^{\prime \prime }$ match with the GK method (see Supplemental Material, Fig. S1 [42]).

The complex modulus, $G^{*}$, related to stress correlation as described in Eq. (1), directly calculated from the simulations for different concentrations are shown in Fig. 3(f). We fit the generalized Maxwell model $G^{*}=\sum_{i}G_{i}\;\text{exp}(-t∕\tau_{i})$ with the complex modulus $G^{*}$ data from the GK method, which matches well with the OS data and scaling exponent follows, at ω→0, $G^{\prime }∼\omega^{2}$, and $G^{\prime \prime }∼\omega$ as shown in Fig. 3(e). In the inset of Fig. 3(e), we show one case for $\phi_{s}=0$, i.e., aged condensed phase, where we fit the Maxwell model for both OS and GK complex modulus. This shows Maxwell-like behavior, corroborating results by Jawerth *et al*. [15] throughout the solvent concentration range. Due to the highly entangled nature of the dense phase, the mean-squared displacement of the chains is subdiffusive $\langle \Delta r^{2}\rangle ∼t^{1∕2}$ as shown in the Fig. 3(g). As solvent is depleted from the dense phase [Fig. 3(e)], the condensate becomes more viscous. Hence, the crossover regime shifts an order of magnitudes in the ω range, which validates the experimental results [15].

### B. Role of chain rigidity and length

**FIG. 4.**
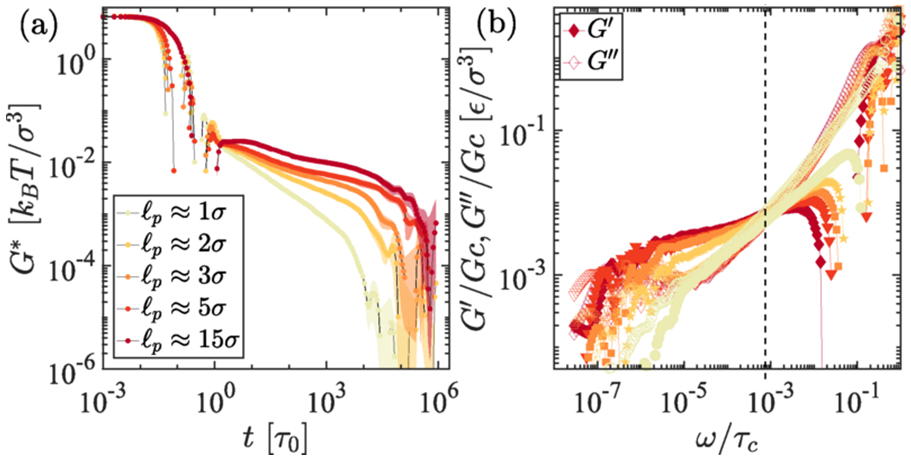
Viscoelastic responses as a function of rigidity of the polymer. (a) Complex modulus from the GK zero shear for different persistence lengths of the chains ranging from fully flexible $\ell_{p}\approx \sigma$ to stiffer chains $\ell_{p}\approx 15\sigma$. (b) The corresponding elastic and viscous moduli, $G^{\prime }$, $G^{\prime \prime }$ from the $G^{*}$.

We capture the intermediate elastic-dominated frequency range of the Maxwell response at a lower shear frequency by introducing an angular potential. In Fig. 4(a), we vary the rigidity of the polymer chains. In a fully flexible chain limit where persistence length is of the order of bead size, i.e., $\ell_{p}\approx \sigma$, the individual chains are in a crumpled state and also are not as entangled as compared to the semiflexible chains [44,45]. While rigidity can influence material properties, it does not directly impact the behavior of condensates when analyzing thermodynamic quantities or constructing phase diagrams. However, in expressing the mechanorheological properties, rigidity plays a crucial role.

We have simulated systems with varying angular potentials to quantify the dependence of the rheological properties of the condensates on biopolymer chain rigidity in solvent-free scenarios. As shown in Fig. 4(b), we show elastic and loss moduli are significantly different between fully flexible chains and rigid chains. We set a range of angles 120°–170° and angular potential $K_{\theta }=10\;k_{B}T$ to probe the rigidity of the polymers. According to the tangent-tangent correlations along the contour length of the chain, and also measure from the equilibrium angle, we calculate the persistent length that set rigidity in each case, which goes from $\ell_{p}=\sigma$ to ∼15σ in Figs. 4(a)-4(b). It is clear from Fig. 4(a), $\ell_{p}>\sigma$ shows multiple relaxation times, which corresponds to an elastic-dominated region in the frequency space. The strengths of angular potential energy can significantly influence the rigidity of the chain, which one can adjust in the simulation setup. Therefore, persistence length calculated from the equilibrium configuration must be used to quantify the rigidity of the chain, $\ell_{p}=-1∕\log(\cos\;\theta_{m})$ (equilibrium angle, $\theta_{m}$ measured from the simulation). Nonetheless, to check consistency with GK, we also perform OS on the same system, probing various conditions such as the equilibrium angle $\theta_{0}=111.6^{{}^{\circ}}$ (flexible), $\theta_{0}=125^{{}^{\circ}}$ $\theta_{0}=150^{{}^{\circ}}$, with $K_{\theta }=10\;k_{B}T$ (semiflexible limit) and $K_{\theta }=0.1\;k_{B}T$ (more flexible) $K_{\theta }=0$ (which has no angular potential i.e., fully flexible chains). It is clear from both GK Fig. 4(b) and OS simulations (in Supplemental Material, Fig. S2(a) [42]) that semiflexible chains show distinct frequency-dependent features of the rheology. At low frequency and high frequencies, viscous modulus dominates, and in the intermediate, elastic modulus dominates in the interim regime $10^{-6}≲\omega ≲5\times 10^{-4}$ resembles Maxwell fluidic nature. However, viscous modulus dominates for flexible chains irrespective of the applied OS rates. One would also be interested in finding the structural properties of the molecules, which can be deciphered by measuring the radius of gyration $\langle R_{g}\rangle$ of the chains. The radius of gyration is higher for polymers with higher rigidity; this corroborates the wormlike polymer chain properties in the semiflexible rigidity limit $\sigma <\ell_{p}\ll L$, $\langle R_{g}\rangle ∼\ell^{0.41}$, which interestingly unravels the intermediate scaling exponent of semiflexible polymer in a dense state (see Supplemental Material, Fig. S2(b) [42]) [46]. However, for the fully flexible polymers, $R_{g}$ is in the range of self-avoiding chain limit; chains are primarily in the collapse state. Therefore, there is less obstruction due to the minimal entanglements in the system. Hence, the rheological properties show fluidic-type distinct features. On the other hand, the semiflexible chains experience significant entanglements that arrest the system, leading to a dominant elastic response region in the OS frequency space [47,48].

**FIG. 5.**
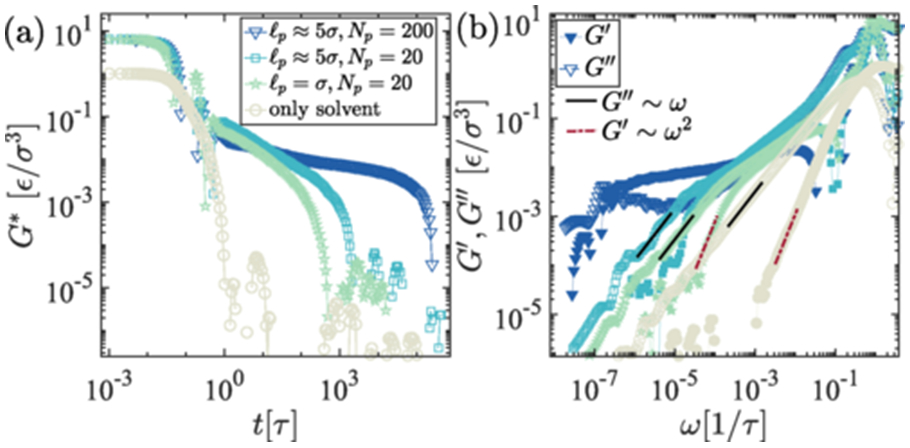
Comparison of viscoelastic responses for different polymer chain lengths, $N_{p}=200$ (with flexibility $\ell_{p}\approx 5\sigma$), $N_{p}=20$ (with flexibility $\ell_{p}\approx 5\sigma$ and σ), and monomers. (a) The complex modulus $G^{*}$ displays faster decorrelation for polymer melts comprising shorter chains, attributed to reduced entanglements and lower friction. (b) Loss and storage modules demonstrate the dominance of the viscous modulus in shorter, more flexible chains compared to longer semiflexible chains.

Biomolecules generally have a more extended backbone, facilitating unique nonequilibrium features inside the condensates. To identify the chain-length-dependent viscoelastic feature, we measure and compare the viscous and loss moduli of the shorter molecules. Chain length plays a crucial role in depicting the viscoelastic response of the condensates. In the earlier measurements, we fix the chain length $N_{p}=200$; however, in Fig. 5, we compare the viscoelastic response to the order of magnitude of smaller chain contour length, i.e., $N_{p}=20$, its stiffness and with monomers. It is evident from the GK measurement in Fig. 5(b) that the shorter chain is unable to exhibit a dominant elastic modulus region in the frequency space. Viscoelastic response primarily shows the viscous behavior of such smaller chain systems. For shorter chains, complex modulus $G^{*}$ are uncorrelated faster compared to the longer chains [in Fig. 5(a)]. It is clear from Fig. 5(b) that the shorter chains also follow the Maxwell fluidic nature at slow deformations.

### C. Application to aging of FUS and PGL-3

**FIG. 6.**
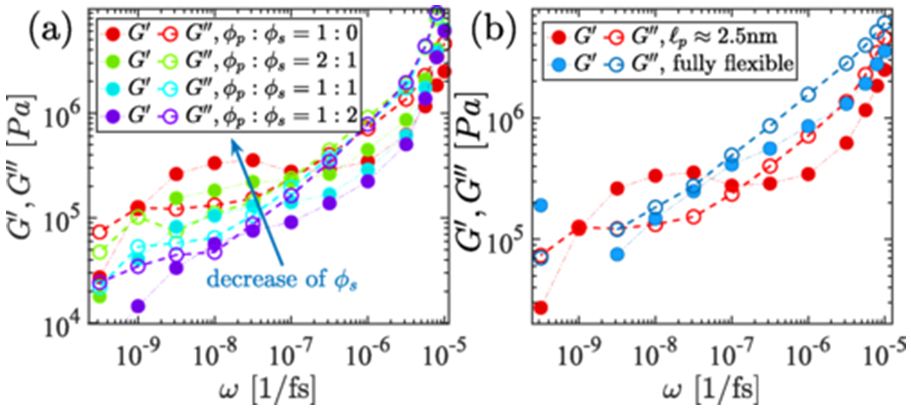
Elastic and loss modulus of FUS with $N_{p}=200$ CG HPS residue model chains are depicted. In (a), the viscoelastic moduli $G^{\prime }$ and $G^{\prime \prime }$ are presented for various solvent concentrations. The elastic response becomes more pronounced as the solvent evaporates. Overall, the observed behavior, both in the presence and absence of solvent, aligns well with a simple LJ bead model. (b) emphasizes the dominance of the elastic response regime with those of semiflexible chains (depicted in red), contrasting fully flexible chains (depicted in blue) while fixing other parameters (both are simulated at $\phi_{s}=0$). For fully flexible chains, viscous modulus dominates throughout the applied strain range.

Here, we implement the microrheology techniques introduced in the previous section in the coarse-grained HPS model of FUS protein to study the rheological properties of the FUS biomolecular condensates. We took a similar volume fraction of the FUS protein and solvent as in the case of LJ systems and observed similar rheological behavior as shown in Fig. 3(e) through the implementation of OS. As solvent fraction $\phi_{s}$ decreases, the elastic modulus-dominated region is more pronounced. In the smaller frequency region ω→0, the viscous modulus $G^{\prime \prime }$ dominates and goes $G^{\prime \prime }∼\omega$ whereas the elastic modulus $G^{\prime }∼\omega^{2}$, replicates the LJ systems. The low-frequency crossover shifts towards lower frequency as solvent egresses the system, as seen in Fig. 6(a). We observed a similar qualitative behavior to that of a generalized Maxwell fluid in the disordered part of the PGL-3 protein using the GK method (see Supplemental Material, Fig. S3 [42]). Our findings indicate that desolvation increases the intermediate elastic-dominated regime and a shift toward longer relaxation time in the viscous crossover with respect to angular frequency (ω) (see Supplemental Material Figs. S3 and S4 [42]). In addition to similar solvent fraction-dependent rheological outcomes, we also observe stiffness dependence viscoelastic behavior that is precisely analogous to LJ (Lennard-Jones) systems, as shown in Fig. 6(b). A fully flexible FUS system (i.e., without the angular potential) is dominated by the viscous response throughout the frequency range. However, adding an angular potential $\ell_{p}=5\sigma$ becomes semiflexible FUS chains, which clearly shows the elastic modulus-dominated regions in Fig. 6(b). Therefore, we conclude that angular potential plays a significant role in demonstrating the viscoelastic behavior of both CG models. Precise nonbonded interactions may play an essential role in defining equilibrium properties where the angular potential is insignificant, as most previous studies have overlooked the role of angular potential. However, to fully comprehend nonequilibrium properties, the angular potential is critical.

### D. Limit of long sticker-sticker lifetimes

**FIG. 7.**
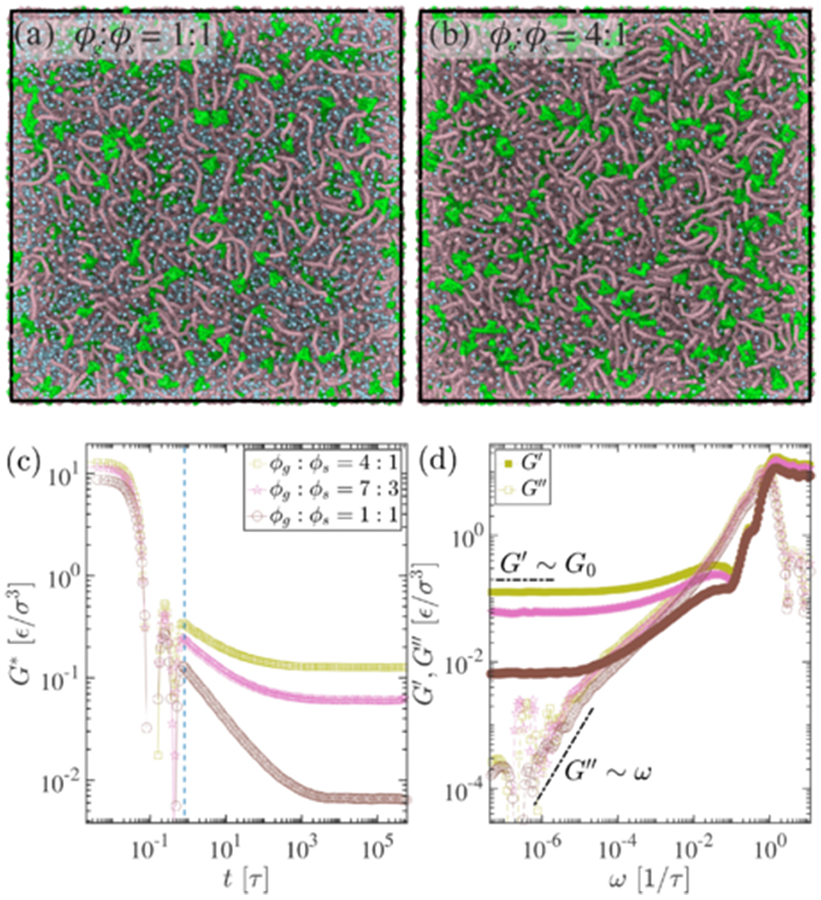
(a), (b) Simulation snapshots of the cross-linked gel network, where polymers of the network ($\phi_{g}$) are denoted by maroon and greenish beads represent the linking elements where the solvent ($\phi_{s}$) is depicted in yellow. Viscoelastic measurements of the cross-linked gel are presented in (c) and (d). In contrast to an uncross-linked polymer, the complex modulus $G^{*}$ of the cross-linked gel stabilizes at a plateau as t→∞ shown in (c). Unlike in a polymeric melt, the elastic modulus $G^{\prime }$ remains independent of the applied frequency at lower frequencies, ω→0, revealing a Kelvin-Voigt solid behavior in (d).

The aging process is associated with the transition of biomolecular condensates from a liquidlike behavior to a gel-like state as the intermolecular network of β sheets becomes more interconnected. This transition restricts the long-term self-diffusion of proteins within the condensates. We employ the GK relation and OS techniques on cross-linked network systems to unravel generic gel system rheological characteristics, which is crucial in the biomolecular aging context. We take different volume fractions $\phi_{g}$ of gel and $\phi_{s}$ solvent as shown in Figs. 7(a)-7(b) simulation snapshot. Note that a higher volume fraction of gel corresponds to an increased number of connectivity nodes present in the system and, consequently, higher rigidity. Unlike un-cross-linked fluid systems in earlier discussions Fig. 3(e), here we see distinct quantitative features of shear moduli Figs. 7(c)-7(d). At lower frequency ω→0, similar to the previous case, viscous modulus $G^{\prime \prime }∼\omega$; however, the elastic modulus $G^{\prime }$ reaches a plateau at $G_{0}$, which is the dominant modulus and independent of ω. This characterizes the Kelvin-Voigt viscoelastic solid model and corroborates aging phenomena reported in Alshareedah *et al*. [16]. Therefore, solvent expulsion brings sticky biopolymer regions closer, resulting in transient network formation in the biomolecular condensates. Although these condensed phases exhibit fluidic properties initially, solvent evaporation facilitates the formation of interconnected β-sheet networks over a prolonged period, resulting in kinetic arrest. This assertion is consistent with the observed reversible hydrogel behavior in specific RNA-binding proteins characterized by a high percentage of β sheets.

### E. Impact of transient sticker-sticker lifetimes

**FIG. 8.**
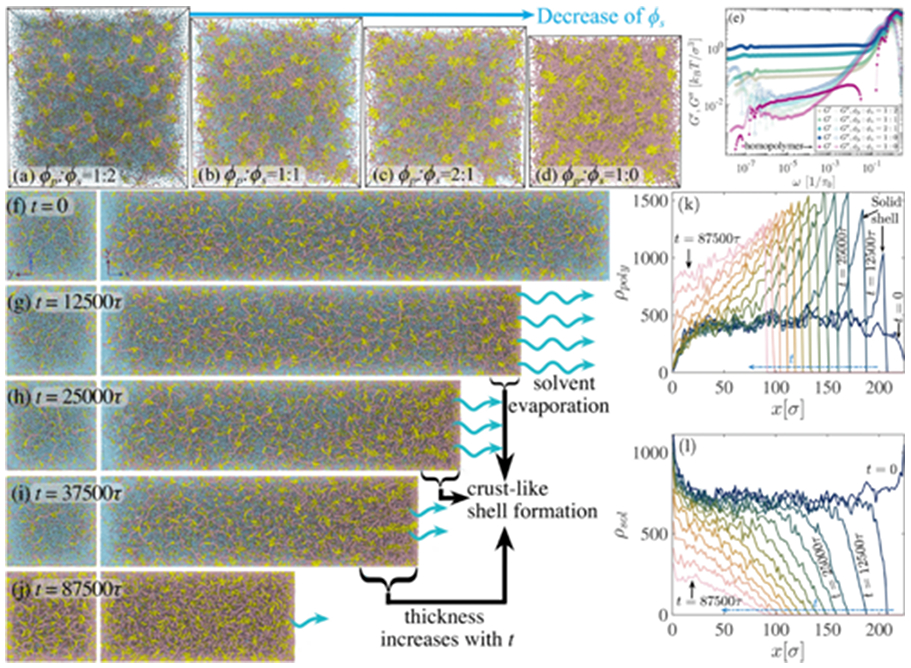
(a)–(d) Simulation snapshots depict the ratio of chains $\phi_{p}$ (represented by pale chestnut-colored strands and yellow stickers) as a function of the decrease in solvent concentration $\phi_{s}$ (depicted by turquoise beads). (e) Storage ($G^{\prime }$) and loss ($G^{\prime \prime }$) moduli for different concentrations of the sticky-rigid patch chains with varying $\phi_{s}$. The homopolymer solution case from Fig. 3(e) is included here as a reference for comparison. (f)–(j) Snapshots from nonequilibrium simulations of aging with evaporating solvent. Two perspective views of the simulation are shown: The left panel displays the squared surface perpendicular to the x direction (nonperiodic boundary) at x(L(t)) where evaporation takes place, while the right panel shows the elongated rectangular direction where the box length is shrinking, forming a crustlike shell at the boundary with time. (k) The number density of the polymer $\rho_{\text{poly}}$ and (l) number density of the solvent $\rho_{\text{sol}}$ in the box along the x direction as a function of time are also presented.

Earlier, we discussed the minimal chain model, where we only studied the effects of desolvation on aging. We demonstrated that to capture the extreme of Kelvin-Voigt solid behavior, one has to introduce sticker pairs with infinitely long lifetimes, which we rationalized in simulations of cross-linked gel-like condensates. Here, we delve into a more general scenario where transient sticker pairs form a thermoreversible network. We conducted simulations with four different solvent concentrations in Figs. 8(a)-8(d) (with volume fractions identical to the previous setup). We placed stickers at fixed ten-bead intervals along the chain. Condensates are thus stabilized by the formation of transient nodes. At higher solvent concentrations, there are more nodes, with a lower sticker number density in each node. As solvent leaves the system, neighboring nodes combine, resulting in higher sticker density in each node. This makes the system more solidlike, mimicking maturation or aging. As seen in Fig. 8(e), the intermediate elastic modulus increases as $\phi_{s}$ approaches zero, while the region of crossover between elastic and viscous moduli increases by orders of magnitude, corroborating experimental results [15]. Note that, as shown in Fig. 8(e), the elastic modulus increases by more than two orders of magnitude when compared with minimal coarse-grained homopolymer systems. Therefore, we infer that sticker motifs play a crucial role in the viscoelastic properties of biomolecular condensates.

### F. Formation of corona shells during maturation

A recent study has reported on the maturation process of liquid droplets at both macroscopic solidification and fibrilization, the mechanism underlying the transition from liquid to solid state by forming a thin crust shell [34,35,49-53]. Spectroscopic observations establish a mechanism for forming a solid shell barrier around the droplets, suggesting that a previously undetected population of FUS at the droplet surface gradually converts into a solid crust that influences protein exchange.

Aging of condensate can take days a time scale, which is not easily accessible within a single equilibrium molecular dynamics simulation. To this end, we have set up nonequilibrium simulations to model condensate aging resulting from an interplay of protein chain rigidification, desolvation, and sticker pair formation time scales. Our setup involves a cuboid box elongated in the x direction, with fixed boundaries along the x direction and periodic boundary conditions implemented along the orthogonal directions y and z. Desolvation occurs at x(L) with a fixed rate, expelling only solvent molecules at the boundary [see Figs. 8(f)-8(j)]. Solvent molecules located near the surface between the simulation box ($\Delta x=10^{-2}\sigma$) can evaporate at a rate of ten solvent atoms in a unit time τ. The expulsion of solvent from the surface causes the box to shrink and sticky regions to converge, forming a polymer-dense phase $\rho_{\text{poly}}$ at the corona region [Figs. 8(k) and 8(l)]. Over time, we observe biopolymer shell growth mimicking solidlike corona seen in experiments [34]. Furthermore, the pores that allow solvent permeation from inside of the condensed phase shrink over time, slowing down the rate of desolvation and eventually trapping solvent inside the condensate (see Supplemental Material, video 1 [42]). These pore sizes and closer rates are sensitive to sticker interaction $\chi_{\text{ps}}$ and the rigidity of the chain. Simulations with stronger sticker interactions lead to faster solvent entrapment in the condensed phase, which leads to denser corona shells, leading to a dramatic slowdown of the aging process (see Supplemental Material, video 2 [42]).

## IV. DISCUSSION

Recent experiments [15,17,23,34] report that the aging of biomolecular condensates is likely accompanied by solvent expulsion, the formation of rigid structural elements, and percolated sticky regions [54-60]. However, the detailed interplay of all these factors and the associated time scale on the dynamics of aging has remained poorly understood. In the present study, we have systematically dissected the distinct contributions of chain rigidification, chain length, desolvation, and formation of sticker linkages. Most importantly, we have shown that the interplay of all these factors can account for Maxwell fluid and Kelvin-Voigt solid phase formation during the maturation process. Furthermore, we demonstrate that an interplay of time scales associated with desolvation and sticky pair formation can recapitulate the recently observed corona shell formation accompanying the aging of condensates [23,34]. Below, we provide a brief description of our findings.

First, we have established a necessary condition to observe viscoelasticity in typical residue-level simulations of condensates. We find that fully flexible chains commonly used in models of intrinsically disordered proteins, regardless of their sequence-specific interactions, consistently exhibit a viscous-dominated behavior across all frequencies. On the other hand, after accounting for rigidity and making chains semiflexible, we observe generalized Maxwell-type behaviors involving viscous and elastic components across various applied shear frequencies. This aligns with recent experimental results [15] and highlights the importance of accounting for the semiflexible aspect of intrinsically disordered proteins in future studies.

We observe significantly different responses when comparing the viscoelastic response of long chains with shorter chains. Shorter chains, with fewer entanglements, predominantly exhibit viscous behavior, while the intermediate elastic response disappears entirely due to reduced entanglement effects. We show that desolvation is a significant factor contributing to the aging of condensates. This behavior can be attributed to two factors: In the early stages of condensation, the condensed phase exhibits predominantly viscous behavior with minimal overlap in the elastic regions $G^{\prime }$ and $G^{\prime \prime }$ [61,62]. In the later stages (typically after 48 h), the solvent evaporates from the condensed phase, leaving behind a protein-rich phase that increases the system’s viscosity. We report a prominent elastic-dominated regime in the frequency space and a viscous-dominated regime at high and slow deformation rates.

We explain the viscoelastic behavior of biomolecular condensates at different aging states through an interplay of sticker lifetimes. The experiments by Jawerth *et al*. have reported a Maxwell fluid behavior [15], which is recapitulated in our simulations by forming transient thermoreversible sticker pairs. The experiments by Alshareedah *et al*. [16] have reported another aging extreme, better described as Kelvin-Voigt solid response. We show that this aging stage can be explained by the long lifetimes of stickers, which results in an effectively cross-linked polymeric gel network. Unlike long entangled chains, chemically cross-linked gels percolate through the box and provide a constant complex modulus $G^{*}$ in the limit of long times.

Finally, we have incorporated the key molecular processes associated with aging—chain rigidity, desolvation, and sticker pair formation—into a nonequilibrium dynamic simulation to simulate the aging process explicitly. An intricate interplay among these molecular events collectively contributes to the formation of a solidlike shell around the condensate surface. This shell formation and solvent entrapment within aged condensates align with recent experimental reports [23,34,35]. We believe that through this nonequilibrium mechanistic exploration of aging, we offer a comprehensive mechanistic insight into the molecular underpinnings of aging in condensates, paving the way for further exploration in this field.

## Supplementary Material

Supplementary materials

SI Video 1

SI Video 2
