# Supplementary material for: Molecular Drivers of Aging in Biomolecular Condensates: Desolvation, Rigidification, and Sticker Lifetimes
Source: PRX Life. Author manuscript; Available in PMC 2025 Jan 25. (PMC11759064; doi:10.1103/prxlife.2.023011)
Supplement: Supplementary materials [file NIHMS2008027-supplement-Supplementary_materials.pdf]

SI: Molecular Drivers of Aging in Biomolecular Condensates: Desolvation, Rigidification, and Sticker Lifetimes

Subhadip Biswas[1*] and Davit A Potoyan[1,2†]
[1]Department of Chemistry, Iowa State University, Ames, IA 50011, USA and
[2]Department of Biochemistry, Biophysics and Molecular Biology, Iowa State University, Ames, IA, 50011, USA
(Dated: May 20, 2024)

A. Linear viscoelastic regime

Mechanical properties of the viscoelastic materials crucially depend on applied strain amplitude. Typically, a small amplitude of the oscillatory shear exhibits linear rheology. In simulations, this linear viscoelastic (LV) region is typically identified through amplitude sweeps, where the storage modulus (G') and loss modulus (G'') are plotted against applied strain amplitude. We conducted the OS simulations in various strain amplitudes, γ_0 (Fig (1 a)). As shown in Fig (1 b), the plateau region represents the LV region. Within the LV region, both G' and G'' remain relatively constant, which signifies that the material's response to mechanical deformation is proportionate and follows linear rheology. It is noted that the lower stress amplitude matches the GK shear modulus calculations well. As seen from Fig (1 a), at higher frequencies, a crossover between elastic and loss moduli shift from crossover A to B by increasing the γ_0. The second crossing points at lower frequencies are identical at the same ω_c for all the applied strain amplitudes.

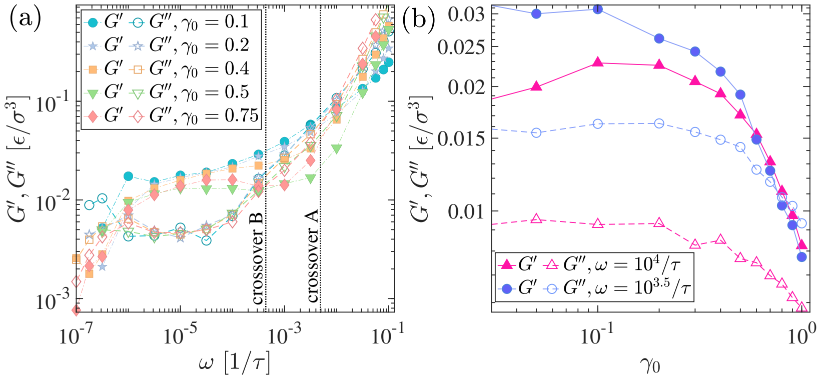

FIG. 1. Viscoelastic responses are observed as a function of shear amplitude using OS (in the case of $\ell_p = 5\sigma$). In the limits of $\omega \to 0$, the shear amplitude γ_0 exhibits similar behavior, showing a consistent crossover point from the elastic to the viscous-dominated region. However, at higher shear rates, G' and G'' demonstrate significantly different responses, with the crossover between the viscous and elastic-dominated regions shifting from A (for $\gamma_0 \leq 0.2$) to B for higher shear rates ($\gamma_0 > 0.2$) as shown in (a). The linear viscoelastic region as a function of shear amplitude γ_0 is depicted in (b). For lower γ_0, G' and G'' remain constant and decay as the amplitude increases.

B. Impact of chain rigidity of biopolymers:

Change in rigidity impacts the viscoelastic properties of the polymer melt. We calculate the equilibrium average angle of the homopolymer chains from the simulation and, hence, the persistence length from the formula: $\ell_p = -1/\log(\cos\theta)$. Here, θ is the angle between three consecutive beads of the chain. Harmonic angular potential is used to fix the angle between consecutive CG beads. The value of the angular potential K_θ is set to be constant throughout the simulations. However, note that choosing the value of K_θ can renormalize the persistence length of the chain in

* subhadip@iastate.edu
† potoyan@iastate.edu

a typical polymer melt. As depicted in Fig. (2 a), we vary the angular potential and provide an equilibrium angle θ_0. Chain conformation sets an equilibrium angle in the lowest energy state of the bulk systems. We calculate the equilibrium angle using the aforementioned equation, validating the length over which the tangent correlation $\langle \vec{r}_i \cdot \vec{r}_j \rangle$ approaches zero.

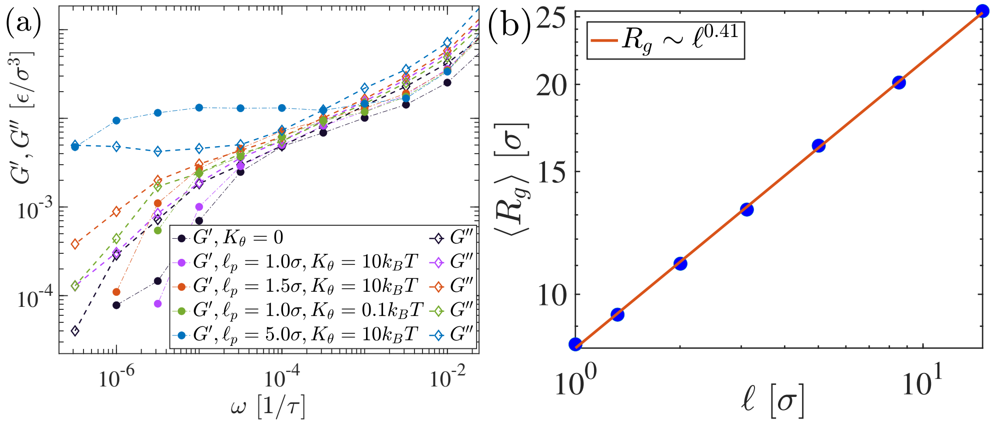

FIG. 2. Viscoelastic responses as a function of rigidity of the polymer are shown in (a). For fully flexible polymer systems, viscous modulus dominates throughout the frequency range of ω. In contrast, introducing rigidity changes the rheological properties significantly. An elastic-dominated frequency range emerges as polymer rigidity increases. In (a), $G'\&G''$ from OS are shown for different stiffness conditions of the chain. Only persistence length $\ell_p = 5\sigma$ shows elastic-dominated characteristics. The radius of gyration $\langle R_g \rangle$ for different stiffness of the chains are shown in (b).

As seen from Fig. (2a), in the oscillatory deformation simulation, a chain with significant persistence length can only exhibit an intermediate elastic-dominated regime in the deformation frequency ω. Fully flexible chains or chains with lower persistence length cannot express an elastic-dominated response. In this scenario, reptation occurs within a lower relaxation time, indicating chains must have different conformational properties. This can be seen in Fig. (2b) where we show the radius of gyration has scaling behavior as a function of persistence length $\langle R_g \rangle \sim \ell_p^\alpha$. We extrapolate the exponent $\alpha \approx 2/5$. This result indicates that fully flexible chains have fewer contacts with neighboring chains and form a collapsed state. As we increase the chain stiffness, the number of contacts increases; hence, reptation takes a longer time and shows generalized Maxwell behavior.

C. Viscoelastic properties of FUS and PGL-3 with desolvation using Green-Kubo

A disorder part of PGL-3 sequence we have taken:

500 510 520 530 540 550 560

DLANVLNSAKIIGESVTVAAHDVIPEKLNAEKNDNTPSTASPVQFSSDGWDSPTKSVALPPKISTLEEEQEEDTT 568

570 580 590 600 610 620 630 640

ITKVSPQPQERTGTAWGSGDATPVPLATPVNEYKVSGFGAAPVASGFGQFASSNGTSGRGSYGGGRGGDRGGRGA 643

650 660 670 680 690

YGGDRGRGGSGDGSRGYRGGDRGGRGSYGEGSRGYQGGRAGFFGGSRGGS 693

A disorder part of FUS sequence we have taken:

10 20 30 40 50 60 70

MEANKRQIVEVDGIKSYFFPHLAHYLASNDELLVNNIAQANKLAAFVLGATDKRPSNEEIAEMILPNDSSAYVLA 75

80 90 100 110 120 130 140 150

AGMDVCLILGDDFRPKFDSGAEKLSQLGQAHDLAPIIDDEKKISMLARKTKLKKSNDAKILQVLLKVLGAEEAEE 150

160 170 180 190 200

KFVELSELSSALDLDFDVYVLAKLLGFASEELQEEIEIIRDNVTDAFEAC 200

FUS and PGL-3 have primarily been used in recent experimental studies to observe aging or the maturation of protein condensates. Therefore, we adopt the HPS model under explicit solvent conditions to achieve similar rheological properties. With an increase in polymer concentrations, the elastic modulus significantly increases. However, it is important to note that long-time behavior is challenging to capture even within the current HPS residue CG simulation framework due to computational limitations. One must consider running simulations for extended periods with multiple independent runs to reduce errors and accurately capture the behavior of $G' \sim \omega^2$ and $G'' \sim \omega$ as $\omega \to 0$. Nevertheless, both PGL-3 in Fig.(3) and FUS in Fig.(4) exhibit quantitatively similar behavior, as discussed in the main text. We have considered the same chain length $N_p = 200$ for these proteins to maintain consistency throughout this work. As mentioned above, we have only included the disordered portion of these FUS and PGL-3 proteins. Over time, we observed that desolvation affects the viscoelastic properties and prolongs the relaxation time.

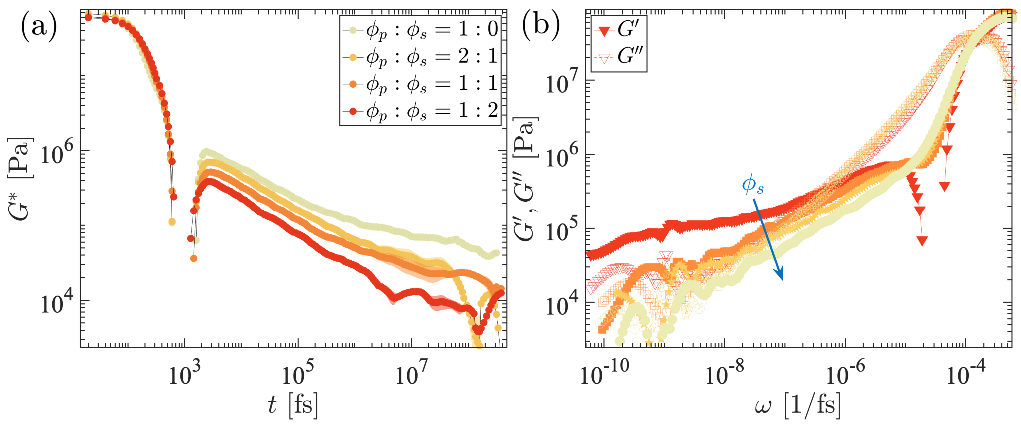

FIG. 3. Elastic and loss modulus of PGL-3 with $N_p = 200$ CG HPS residue model chains are depicted. In panel (a) complex modulus G^*, and (b), the viscoelastic moduli G' and G'' are presented for various solvent concentrations. The elastic response becomes more pronounced as the solvent evaporates. Overall, the observed behavior, both in the presence and absence of solvents, aligns well with a simple LJ bead model.

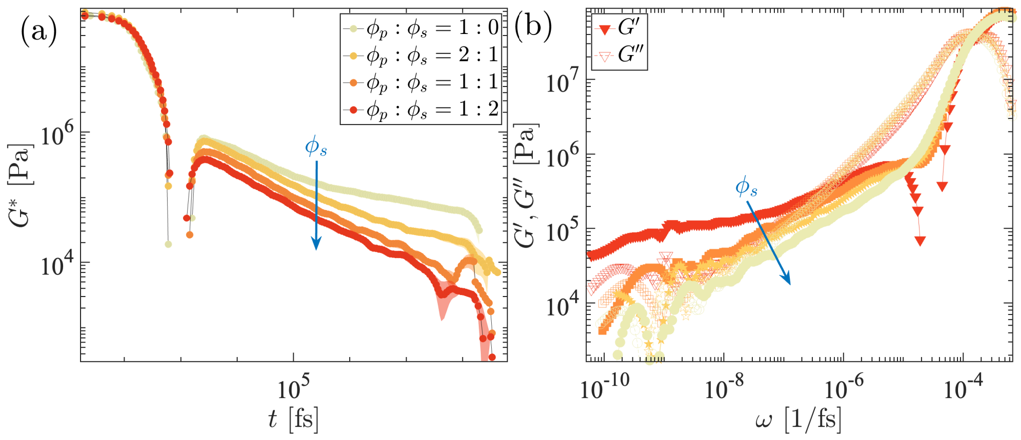

FIG. 4. Elastic and loss modulus of FUS with $N_p = 200$ CG HPS residue model chains are depicted. In panel (a) complex modulus G^*, and (b), the viscoelastic moduli G' and G'' are presented for various solvent concentrations. The elastic response becomes more pronounced as the solvent evaporates. Overall, the observed behavior, both in the presence and absence of solvents, aligns well with a simple LJ bead model.
